# OCT-based vulnerable plaque features and MACE prediction in premature coronary artery disease

**DOI:** 10.3389/fcvm.2025.1621824

**Published:** 2025-10-15

**Authors:** Jiabao Liu, Haoyu Meng, Leilei Chen, Liansheng Wang, Jiayang Xu

**Affiliations:** ^1^ Department of Cardiology, The First Affiliated Hospital with Nanjing Medical University, Nanjing, Jiangsu, China; ^2^ Department of Cardiology, Nanjing Jiangning Hospital of Traditional Chinese Medicine, Nanjing, Jiangsu, China

**Keywords:** premature coronary artery disease (PCAD), optical coherence tomography (OCT), vulnerable plaque, major adverse cardiovascular events (MACE), risk stratification

## Abstract

**Objective:**

This study aimed to systematically analyze coronary plaque characteristics in patients with premature coronary artery disease (PCAD) using optical coherence tomography (OCT) and clarify their associations with clinical risk factors and major adverse cardiovascular events (MACE).

**Methods:**

A total of 224 patients (men ≤55 years, women ≤65 years) with suspected or confirmed CAD who underwent coronary angiography and OCT at the First Affiliated Hospital of Nanjing Medical University between February 2022 and February 2024 were enrolled. Among them, 142 were diagnosed with PCAD (observation group), and 82 had coronary stenosis <50% (control group). Baseline clinical data, risk factors, and OCT-derived plaque features were collected. Patients were followed for 12 months to record MACE. Statistical analyses included independent *t*-tests, chi-square tests, and multivariate Cox regression.

**Results:**

The PCAD group exhibited significantly higher prevalence rates of hypertension (63.38% vs. 47.56%), smoking (30.28% vs. 17.07%), and diabetes (19.72% vs. 8.54%), along with elevated total cholesterol (4.89 ± 1.41 vs. 4.41 ± 1.32 mmol/L), LDL-C (2.91 ± 0.98 vs. 2.51 ± 0.72 mmol/L), and lipoprotein(a) (50.2 ± 28.4 vs. 30.5 ± 18.7 mg/dl) compared to controls (all *p* < 0.05). OCT analysis revealed higher vulnerability in PCAD plaques, characterized by thinner fibrous caps (150.16 ± 82.71 vs. 250.71 ± 123.53 µm, *p* < 0.01), larger lipid arc (93.21 ± 36.43° vs. 60.10 ± 24.46°, *p* < 0.01), increased macrophage infiltration (19.01% vs. 4.87%, *p* < 0.01), and more intraplaque microchannels (14.79% vs. 8.53%, *p* < 0.05). During follow-up, MACE incidence was significantly higher in the PCAD group (12.68% vs. 3.70%, *p* < 0.01). Multivariate Cox regression identified thin-cap fibroatheroma (HR = 2.95), lipid arc ≥180° (HR = 2.61), macrophage infiltration (HR = 1.98), plaque rupture (HR = 2.82), and thrombosis (HR = 2.30) as independent predictors of MACE.

**Conclusion:**

Patients with PCAD demonstrate distinct coronary plaque vulnerability features closely associated with metabolic and lifestyle-related risk factors. OCT enables precise identification of high-risk plaques, providing critical insights for early intervention and risk stratification to mitigate acute cardiovascular events.

## 1 Introduction

Cardiovascular diseases remain the leading cause of death worldwide, with coronary artery disease accounting for nearly two-thirds of cardiovascular-related deaths (1). In recent years, there has been a trend of younger onset of coronary artery disease. Premature Coronary Artery Disease (PCAD), characterized by its distinct pathological features and poor prognosis, has gradually become a focus of clinical research. PCAD is defined as coronary artery disease occurring in relatively younger populations, typically referring to cases developing before age 55 in males and 65 in females, as commonly used in major guidelines and epidemiological studies (2, 3). Studies indicate that PCAD patients frequently present with risk factors such as metabolic syndrome and smoking, and their plaques may exhibit significantly higher vulnerability compared to typical CAD patients. However, the precise pathological mechanisms remain incompletely understood. Optical Coherence Tomography (OCT), with its ultra-high resolution of 10–15 μm, enables precise identification of microscopic plaque characteristics including fibrous cap thickness, lipid core distribution, macrophage infiltration, and microchannels, earning it the designation of “optical biopsy” (4). Multiple studies have confirmed OCT's significant value in identifying vulnerable plaques, guiding interventional therapy, and predicting cardiovascular events (5, 6). Nevertheless, current research on OCT characteristics of coronary plaques in PCAD patients remains scarce, and the relationship between plaque morphology and clinical outcomes remains unclear.

This study aims to systematically analyze coronary plaque characteristics in PCAD patients using OCT technology and investigate their correlations with metabolic risk factors and major adverse cardiovascular events (MACE). The findings are expected to provide imaging-based evidence for early risk stratification, precise intervention, and prognostic evaluation in PCAD patients, thereby reducing the incidence of acute cardiovascular events.

## 2 Methods

### 2.1 Study population

This single-center prospective study was approved by the Ethics Committee of the First Affiliated Hospital of Nanjing Medical University (Approval No.: 2022-SR-529), and written informed consent was obtained from all participants. Premature coronary artery disease (PCAD) was defined as coronary stenosis ≥50% in at least one major epicardial vessel in men ≤55 years or women ≤65 years (2, 3). The inclusion criteria were as follows: (1) Age range: Males ≤55 years, females ≤65 years, consistent with the commonly used definition of premature CAD; (2) Underwent coronary angiography with Optical Coherence Tomography (OCT) at our institution between February 2022 and February 2024; (3) Clinical diagnosis of suspected or confirmed coronary artery disease. Exclusion criteria: (1) Severe hepatic or renal dysfunction (eGFR <30 ml/min/1.73 m^2^ or ALT/AST >3× upper limit of normal); (2) History of coronary artery bypass grafting; (3) Comorbid malignancies or immune system diseases; (4) OCT image quality insufficient for analysis (e.g., incomplete vessel visualization or significant motion artifacts). Clinical indications for coronary angiography included chest pain symptoms and positive non-invasive test results to confirm the presence of coronary stenosis or obstruction. Indications for OCT encompassed angiographically ambiguous or suspicious lesions, vulnerable plaque identification, and non-obstructive coronary artery disease. A total of 224 patients were ultimately enrolled and divided into two groups based on coronary stenosis severity: Observation group (PCAD group, *n* = 142): Coronary stenosis ≥50%, meeting diagnostic criteria for PCAD (males ≤55 years, females ≤65 years); Control group (*n* = 82): Controls were defined as patients with coronary stenosis <50% and no history of acute coronary syndrome. Although these patients underwent angiography and OCT due to clinical suspicion of CAD, they served as a clinically relevant comparator group to evaluate plaque characteristics in a lower-risk population within the same clinical context. This design helps control for referral bias by ensuring both groups were evaluated under similar clinical indications. Routine laboratory tests (complete blood count, routine urinalysis and serum chemistry profile) were performed in the local laboratories of the participating institutions.

### 2.2 OCT procedure and coronary plaque characterization

#### 2.2.1 Equipment and imaging protocol

This study utilized Abbott's latest-generation Optical Coherence Tomography system (OPTIS™ Integrated System, Model: C408661) with a high-resolution Dragonfly™ OPTIS™ intravascular imaging catheter (Model: C408645). The system supports 3D vascular reconstruction and real-time plaque stress analysis, achieving a spatial resolution of 10–15 μm and an axial scan rate of 75 mm/s, significantly reducing motion artifacts and optimizing imaging quality.

#### 2.2.2 Standardized protocol

##### 2.2.2.1 Catheter preparation

Adhering to international OCT imaging guidelines (7), the catheter was activated and flushed with iso-osmolar iodinated contrast to eliminate air bubbles at the tip.

##### 2.2.2.2 Image acquisition

An automatic pullback mode (25 mm/s) was employed, synchronized with a contrast injection system (ACIST CVi®) delivering 4 ml/s (total volume: 8–12 ml) to ensure vascular expansion and continuous imaging.

##### 2.2.2.3 Quality control

Signal intensity was monitored in real time (target ≥7/10), excluding cases with blurred images or incomplete vessel visualization.

#### 2.2.3 Plaque characterization

Based on the EACVI OCT Expert Consensus (8), plaque classification and parameters were defined as follows:

• Vulnerable Plaque: Meeting ≥2 criteria:
  (1) Fibrous cap thickness (FCT) <65 μm;
  (2) Lipid arc ≥180°;
  (3) Macrophage density >10% (quantified via AI algorithm);
  (4) Intraplaque microchannels ≥2.
• Plaque Erosion: Intact fibrous cap with endothelial denudation and adherent thrombus (length >2 mm).
• Calcified Nodule: Calcium protrusion occupying ≥50% of the lumen with overlying cap rupture.

#### 2.2.4 Manual OCT image analysis and quantification

All OCT image analyses were performed manually by two experienced and blinded independent analysts using dedicated offline review software (Abbott OPTIS™ Review Software). Each plaque parameter was measured in accordance with the EACVI OCT Expert Consensus guidelines. The following features were quantitatively assessed:

Fibrous Cap Thickness (FCT): The thinnest part of the fibrous cap covering a lipid plaque was identified in multiple cross-sectional frames and measured three times; the minimum value was recorded (precision: ±5 m).
Lipid Arc: The maximum angular extent of the lipid-rich core was measured in the cross-section with the largest lipid accumulation.
Macrophage Infiltration: Defined as focal, signal-rich regions with high attenuation, typically exhibiting a “bright spot” appearance with backward shadowing.
Intraplaque Microchannels: Identified as well-delineated, signal-poor voids within the plaque, visible on at least three consecutive cross-sectional frames.

Interobserver agreement for all quantitative measurements was evaluated using Bland-Altman analysis, which demonstrated excellent consistency (intraclass correlation coefficient, ICC > 0.90). Discrepancies were resolved by consensus with a third senior analyst.

#### 2.2.5 Data standardization and quality assurance

All images were independently analyzed by two blinded, experienced analysts. Interobserver agreement was validated using Bland-Altman analysis (ICC >0.90).

### 2.3 Study endpoints

#### 2.3.1 Primary endpoint

Major Adverse Cardiovascular Events (MACE), defined as a composite of cardiovascular death, non-fatal myocardial infarction, ischemia-driven revascularization, and ischemic stroke.

#### 2.3.2 Secondary endpoint

All-cause mortality. Each event was adjudicated by two independent cardiologists based on clinical, laboratory, and imaging criteria.

### 2.4 Follow-up

Patients were followed for 12 months via outpatient visits or telephone interviews. Medical records, laboratory results, and imaging reports were reviewed to document any occurrence of MACE or mortality. Follow-up was completed for all enrolled patients.

### 2.5 Adjudication of clinical events

All reported endpoints, including major adverse cardiovascular events (MACE) and all-cause mortality, were prospectively adjudicated by an independent Clinical Event Committee (CEC) comprising two interventional cardiologists and one neurologist. The CEC members were blinded to the patient's group allocation and OCT findings. Event definitions were pre-specified in the study protocol. Myocardial infarction was defined according to the Fourth Universal Definition of Myocardial Infarction. Ischemia-driven revascularization required objective evidence of ischemia on functional testing or imaging concomitant with angiographic stenosis >70%. Stroke was confirmed by neurologist assessment and brain imaging. Discrepancies in adjudication were resolved by consensus.

### 2.6 Statistical analysis

Data analysis was performed using SPSS 26.0. Normality of continuous variables was assessed using the Shapiro–Wilk test (for sample sizes ≤50) or the Kolmogorov–Smirnov test (for sample sizes >50). Continuous variables were expressed as mean ± standard deviation (normally distributed) or median (interquartile range) (non-normally distributed). For variables violating normality assumptions (*p* < 0.05 in normality tests), non-parametric tests or logarithmic transformations were applied prior to analysis. Intergroup comparisons utilized independent t-tests (normal distribution) or Mann–Whitney *U*-tests (non-normal distribution). Categorical variables were reported as frequency (percentage), and group differences were assessed using chi-square tests or Fisher's exact tests (for expected cell counts <5). Multivariable Cox proportional hazards models were applied to identify independent predictors of major adverse cardiovascular events (MACE). Variables with *p* < 0.1 in univariate analyses were included, and results were expressed as hazard ratios (HR) with 95% confidence intervals (CI). Variance inflation factors (VIF) were calculated to evaluate multicollinearity among covariates (VIF >5 indicating significant collinearity). Interobserver agreement was evaluated using Bland-Altman analysis, with intraclass correlation coefficients (ICC) >0.85 indicating good consistency. A two-tailed *p*-value <0.05 was considered statistically significant. Multivariate models included adjustment for referral-related variables (e.g., symptomatic status, non-invasive test results).

## 3 Results

### 3.1 Baseline characteristics

The Premature Coronary Artery Disease (PCAD) group demonstrated significantly higher serum total cholesterol (TC), low-density lipoprotein cholesterol (LDL-C), Lp(a) levels, smoking rates, prevalence of hypertension, diabetes, and family history of coronary artery disease compared to the control group, as shown in Table 1.

**Table 1:** Comparison of baseline characteristics between the premature coronary artery disease group and the control group.

Parameter	Control group (*n* = 82)	Premature CAD group (*n* = 142)	χ^2^/*t*	*p*
Mean age (years)	48.7 ± 5.3	49.3 ± 6.7	0.695	0.488
Male/Female (cases)	58/24	96/46	0.236	0.627
TC (mmol/L)	4.41 ± 1.32	4.89 ± 1.41	2.511	<0.05
LDL-C (mmol/L)	2.51 ± 0.72	2.91 ± 0.98	3.226	<0.01
Lp(a) (mg/dl)	30.5 ± 18.7	50.2 ± 28.4	6.203	<0.01
TG (mmol/L)	2.05 ± 0.87	2.21 ± 1.12	1.114	0.267
HDL-C (mmol/L)	1.31 ± 0.34	1.22 ± 0.43	1.624	0.106
Smoking [*n* (%)]	14 (17.07%)	43 (30.28%)	4.780	<0.05
Hypertension [*n* (%)]	39 (47.56%)	90 (63.38%)	5.326	<0.05
Diabetes (%)	7 (8.54%)	28 (19.72%)	4.930	<0.05
Family history of CAD (%)	5 (6.10%)	22 (15.49%)	4.329	<0.05

Continuous variables are expressed as mean ± standard deviation; categorical variables are presented as number (percentage).

### 3.2 OCT coronary plaque analysis

In the assessment of vulnerable plaque characteristics, the Premature Coronary Artery Disease (PCAD) group exhibited significantly thinner fibrous cap thickness (150.16 ± 82.71 µm vs. 250.71 ± 123.53 µm, *p* < 0.01) and a higher proportion of thin-cap fibroatheroma [TCFA, defined by FCT <65 μm according to consensus standards (8)] (13.38% vs. 2.43%, *p* < 0.01) compared to the control group.

Additionally, the PCAD group demonstrated significantly worse outcomes in lipid-rich plaque prevalence, lipid arc angle, macrophage infiltration, endothelial erosion, plaque fissures, intraplaque microchannels, and thrombus formation. A detailed comparison of plaque characteristics between the control group and PCAD group is presented in Table 2.

**Table 2:** Comparison of plaque characteristics between the premature coronary artery disease group and the control group.

Parameter	Control group (*n* = 82)	Premature CAD group (*n* = 142)	χ^2^/*t*	*P*
Stenosis severity (% ± SD)	35.12 ± 11.34	60.15 ± 21.37	9.831	<0.01
Fibrous cap thickness (µm ± SD)	250.71 ± 123.53	150.16 ± 82.71	7.281	<0.01
Thin-cap fibroatheroma [*n* (%)]	2 (2.43)	19 (13.38)	7.324	<0.01
Fibrous plaque [*n* (%)]	42 (51.22)	45 (31.69)	8.346	<0.01
Calcified plaque [*n* (%)]	17 (20.73)	38 (26.76)	1.020	0.313
Lipid-rich plaque [*n* (%)]	23 (28.05)	59 (41.55)	4.083	<0.05
Lipid arc (° ± SD)	60.10 ± 24.46	93.21 ± 36.43	15.592	<0.01
Macrophage infiltration [*n* (%)]	4 (4.87)	27 (19.01)	8.711	<0.01
Plaque erosion [*n* (%)]	3 (3.65)	22 (15.49)	7.343	<0.01
Plaque rupture [*n* (%)]	0 (0.00)	13 (9.15)	–	<0.01*
Intraplaque microvessels [*n* (%)]	7 (8.53)	21 (14.79)	5.149	<0.05
Thrombus [*n* (%)]	0 (0.00)	9 (6.33)	–	<0.05*

* Fisher's exact test.

We performed a Pearson correlation analysis between LDL-C levels and lipid arc in the PCAD group. The results showed a moderate positive correlation (r = 0.42, *p* < 0.01). Smokers had significantly thinner FCT (132.5 ± 75.2 µm vs. 161.8 ± 86.4 µm, *p* = 0.02).

### 3.3 Representative OCT imaging of coronary plaque morphology

**Figure 1:**
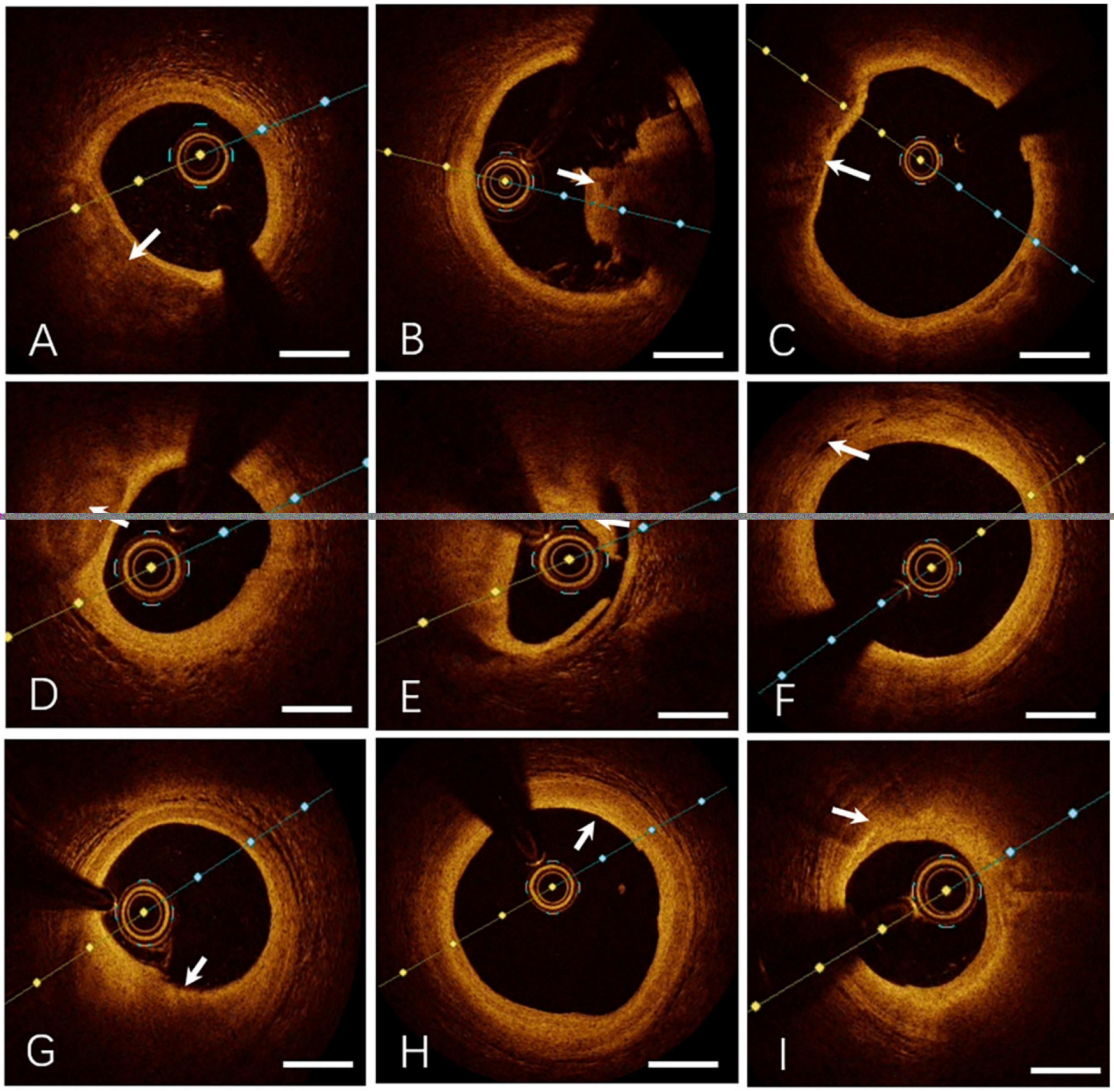
Representative OCT imaging of coronary plaque morphology. **(A)** Lipid-rich Plaque, **(B)** Thrombus (Red Thrombus), **(C)** Thin-cap Fibroatheroma, **(D)** Calcified Plaque, **(E)** Plaque Rupture, **(F)** Intraplaque Microvessels, **(G)** Plaque Erosion, **(H)** Fibrous Plaque, **(I)** Macrophage Infiltration. Specific OCT image features are indicated by the white arrows. Bar = 1 mm.

Figure 1A. Lipid-rich plaque

• Fibrous cap: Focal high reflectivity (hyperintense regions).
• Lipid core: Homogeneous low-intensity zones with high attenuation.
• Ill-defined interface between fibrous cap and lipid core.

Figure 1B. Thrombus

• Irregular luminal mass adherent to the vessel wall or floating freely.
  ○ Red thrombus: High reflectivity, high attenuation, and posterior shadowing (obscuring underlying structures).
  ○ White thrombus: High reflectivity, low attenuation, with preserved visualization of the vessel wall.

Figure 1C. Thin-cap fibroatheroma (TCFA)

• Fibrous cap thickness ≤65 µm.
• Lipid core extending across ≥2 quadrants.

Figure 1D. Calcified plaque

• Sharply demarcated borders.
• Low reflectivity and attenuation.
• Heterogeneous low-intensity regions.

Figure 1E. Plaque rupture

• Disrupted fibrous cap continuity.
• Cavity formation within the plaque.

Figure 1F. Intraplaque microchannels

• Low-intensity, sharply bordered void-like structures.
• Associated with thinner fibrous caps, larger lipid arc angles, and increased prevalence of TCFA.

Figure 1G. Endothelial erosion

• Intact fibrous cap.
• Overlying thrombus secondary to endothelial dysfunction or denudation.

Figure 1H. Fibrous plaque

• High reflectivity and low attenuation.
• Smooth, homogeneous hyperintense regions.

Figure 1I. Macrophage infiltration

• Focal or clustered hyperreflective signals characterized by:
  ○ High reflectivity and attenuation.
  ○ Dot- or streak-like patterns with radial light scattering.
• Predictive of clinical instability.

### 3.4 Major adverse cardiovascular events (MACE), all-cause mortality, and incidence rates

MACE was defined as a composite of cardiovascular death, non-fatal myocardial infarction, ischemia-driven revascularization, and ischemic stroke. During the 12-month follow-up, the PCAD group exhibited a significantly higher total MACE incidence [12.68% (18/142)] compared to the control group [3.7% (3/82)] (*p* < 0.01). Secondary endpoint (all-cause mortality): PCAD group: 1.41% (2/142), Control group: 0% (0/82). No statistically significant intergroup difference was observed (*p* > 0.05). Event type distributions are summarized in Table 3.

**Table 3:** Comparison of MACE and All-cause mortality between the premature CAD group and the control group during 12-month follow-Up.

Event type	Premature CAD group (*n* = 142)	Control group (*n* = 82)	*P*
Cardiovascular death	1 (0.70%)	0 (0.00%)	0.999
Non-fatal myocardial infarction	8 (5.63%)	1 (1.22%)	<0.05
Ischemia-driven revascularization	9 (6.34%)	2 (2.44%)	<0.05
Ischemic stroke	0 (0.00%)	0 (0.00%)	–
Total MACE	18 (12.68%)	3 (3.70%)	<0.01
All-cause mortality	2 (1.41%)	0 (0.00%)	0.532

Intergroup comparisons were performed using the χ^2^ test or Fisher's exact test (when expected event counts <5).

### 3.5 Predictors of MACE events

Multivariable Cox proportional hazards regression was performed to identify independent predictors of major adverse cardiovascular events (MACE). Variables with a univariate association of *p* < 0.1 were included in the model. To comprehensively adjust for potential confounders, the model incorporated baseline covariates with significant intergroup differences (Table 1), including age, sex, lipid profile [total cholesterol, LDL-C, Lp(a)], hypertension, diabetes, smoking status, and family history of coronary artery disease. Multicollinearity among variables was assessed using variance inflation factors (VIF), with all VIF values <3, indicating no significant collinearity. After adjusting for clinical risk factors, the following OCT-derived plaque features remained independent predictors of MACE (Table 4): Thin-cap fibroatheroma (TCFA) (HR = 2.95, 95% CI 1.48–5.88, *p* < 0.01); Lipid arc ≥180° (HR = 2.61, 95% CI 1.25–5.45, *p* < 0.05); Macrophage infiltration (HR = 1.98, 95% CI 1.02–3.85, *p* < 0.05); Plaque rupture (HR = 2.82, 95% CI 1.38–5.76, *p* < 0.01); Thrombosis (HR = 2.30, 95% CI 1.10–4.81, *p* < 0.05).

**Table 4:** Independent predictors of MACE (multivariate Cox regression analysis).

Variable	HR	95% CI	*P*
Thin-cap fibroatheroma	2.95	1.48–5.88	<0.01
Lipid arc ≥180°	2.61	1.25–5.45	<0.05
Macrophage infiltration	1.98	1.02–3.85	<0.05
Plaque rupture	2.82	1.38–5.76	<0.01
Thrombosis	2.30	1.10–4.81	<0.05

## 4 Discussion

Previous studies have established that PCAD is associated with aggressive plaque phenotypes and poor outcomes. However, detailed OCT-based characterization of plaque vulnerability in this population remains limited. Our study provides a comprehensive analysis of OCT-derived high-risk features in PCAD patients and demonstrates their strong association with metabolic risk factors and 12-month MACE. Furthermore, we identify specific OCT predictors of events, supporting the use of intravascular imaging for risk stratification in young patients.

Coronary artery disease (CAD), the leading cause of cardiovascular mortality worldwide, has shown a rising trend of earlier onset. Premature coronary artery disease (PCAD), characterized by its distinct pathological features and poor prognosis, has become a key focus of clinical research. This study systematically analyzed coronary plaque characteristics in PCAD patients using optical coherence tomography (OCT), revealing significant associations with metabolic risk factors and major adverse cardiovascular events (MACE). MACE in this study was strictly defined as cardiovascular-specific endpoints to more precisely reflect the relationship between plaque vulnerability and acute cardiovascular events. The lack of significant intergroup differences in all-cause mortality (a secondary endpoint) suggests that the high-risk profile of PCAD patients is primarily concentrated in cardiovascular-specific events.

Fibrous cap thickness (FCT) is a critical determinant of plaque stability. In this study, the PCAD group exhibited significantly lower mean FCT and a higher proportion of thin-cap fibroatheroma (TCFA) compared to controls. We used the <65 μm cutoff for TCFA definition as it remains the most widely validated and consensus-recommended value for identifying plaques at the highest risk of rupture, irrespective of patient age (8). This threshold is prognostically significant, as plaques with FCT <65 μm are associated with a substantially increased risk of future MACE. While the mean FCT in our PCAD group was higher (150.16 ± 82.71 μm), the critical finding is the significantly higher prevalence of TCFA (FCT <65 μm) in PCAD patients compared to controls (13.38% vs. 2.43%). This indicates that although the average cap may be thicker, a subset of PCAD patients harbor these very high-risk, rupture-prone lesions. This dichotomy underscores the value of OCT in identifying this high-risk subset within a broader population that may otherwise appear similar based on age or traditional risk factors alone.Thin fibrous caps are prone to shear stress, leading to lipid core exposure and subsequent platelet aggregation and thrombosis. Recent studies have further elucidated the interplay between FCT and the local inflammatory microenvironment: macrophages degrade collagen fibers via matrix metalloproteinases (MMP-2, MMP-9), while oxidized low-density lipoprotein (ox-LDL) activates the NLRP3 inflammasome, promoting IL-1β release and establishing a “inflammation-fibrous cap degradation” positive feedback loop (9). A 2023 OCT-based longitudinal study reported that patients with baseline FCT <65 µm had a nearly fourfold increased risk of plaque rupture within one year, with a post-rupture MACE rate as high as 32% (10). These findings underscore the importance of dynamic FCT monitoring and provide a rationale for intensive lipid-lowering therapy.

Our results show that PCAD patients exhibit more vulnerable plaque features—thinner fibrous caps, larger lipid arcs, and higher rates of macrophage infiltration and thrombosis—than age-matched controls. These findings are consistent with, yet more pronounced than, those reported in the CLIMA study (11) which identified FCT <75 μm, lipid arc >180°, MLA <3.5 mm^2^, and macrophage infiltration as predictors of cardiac events in an older cohort. Notably, our PCAD group demonstrated a higher prevalence of individual high-risk features, underscoring a more aggressive plaque phenotype likely driven by metabolic and inflammatory factors prevalent in younger patients. Together, these findings highlight the utility of OCT in risk stratification across age groups and support its role in guiding early intervention in high-risk PCAD patients.

This study also found a significantly higher incidence of plaque erosion in the PCAD group, often accompanied by thrombus formation. Unlike plaque rupture, erosion is characterized by endothelial denudation with mural thrombus and is more common in younger patients and women. Emerging evidence suggests that plaque erosion may be linked to endothelial dysfunction and vasospasm, driven by reduced nitric oxide (NO) bioavailability and elevated endothelin-1 (ET-1) levels (12). The EROSION III study demonstrated that OCT-guided reperfusion strategies safely reduced stent implantation rates by 15%, with 86% of erosion cases and 41% of rupture cases avoiding stenting (13). Macrophage infiltration, a hallmark of inflammation, further influences plaque stability through phenotypic polarization (pro-inflammatory M1 vs. anti-inflammatory M2). Single-cell sequencing recently revealed that M1 macrophages account for 72% of plaque macrophages in PCAD patients—significantly higher than in older patients—and correlate positively with MMP-9 and TNF-α expression (14). M1 macrophages exacerbate oxidative stress via pro-inflammatory cytokines, while M2 macrophages promote tissue repair. Additionally, intraplaque microchannels, imaging markers of neovascularization, are closely associated with lipid core expansion and intraplaque hemorrhage. Studies report that microchannel density >5 mm^2^ increases intraplaque hemorrhage risk by 3.5-fold and accelerates progression to complex lesions (e.g., rupture or calcified nodules) (15). Such plaques exhibit higher vulnerability, with increased risks of intimal tearing, rupture, TCFA formation, macrophage clustering, and luminal thrombosis, alongside elevated slow-flow rates post-stenting (16). These findings position microchannels as biomarkers for plaque progression and potential targets for anti-angiogenic therapies. Thrombus, a direct manifestation of acute thrombosis, may obstruct the lumen or induce distal embolism, leading to myocardial infarction or stroke. Its predictive value in this study aligns with prior research, reinforcing the importance of antithrombotic therapy in high-risk plaque management.

The PCAD group exhibited significantly higher rates of hypertension, smoking, diabetes, and dyslipidemia, highlighting metabolic dysregulation and adverse lifestyles as key drivers of plaque vulnerability. Chronic hypertension increases vascular shear stress, impairing endothelial function and promoting LDL infiltration into the subintima, where oxidation activates monocyte-to-macrophage differentiation and foam cell formation (17, 18). In this study, stenosis severity correlated positively with hypertension prevalence. Previous studies further demonstrated that intensive blood pressure lowering significantly reduces plaque lipid content, increases FCT, and decreases macrophage density (19, 20), supporting its role in delaying plaque progression. Elevated total cholesterol (TC) and LDL-C levels directly correlate with lipid arc enlargement. Lipid core expansion not only increases plaque volume but also amplifies local inflammation via pro-inflammatory cytokine release. PCSK9 inhibitors have been shown to reduce plaque lipid volume and macrophage density after 12 months of treatment (21, 22). Novel triglyceride (TG)-targeted therapies, such as APOC3 inhibitors, simultaneously lower TG and suppress microchannel formation (23), offering new avenues for personalized lipid management.

Smoking induces reactive oxygen species (ROS) generation, accelerating endothelial apoptosis and collagen degradation while upregulating MMPs to destabilize fibrous caps. In this study, smoking rates were significantly higher in the PCAD group. Persistent smoking attenuates statin-induced plaque stabilization and increases TCFA incidence, emphasizing smoking cessation as critical for long-term prognosis post-PCI in ACS patients (24). Diabetes drives macrophage polarization toward the pro-inflammatory M1 phenotype via insulin resistance (25). Recent studies show that hyperglycemia activates DNA methyltransferases (DNMTs), inducing hypermethylation of anti-inflammatory gene promoters (e.g., KLF4) and perpetuating inflammation (26), suggesting that glycemic control may modulate plaque phenotypes through epigenetic mechanisms.

This study has several limitations. First, the single-center design may limit the generalizability of our findings, although the use of standardized OCT imaging and analysis protocols strengthens internal validity. Second, the follow-up duration was limited to 12 months, which may not capture long-term cardiovascular outcomes or delayed plaque progression. Third, our predictive model for MACE lacks external validation in an independent cohort, which is necessary to confirm its broader applicability. Future multicenter studies with extended follow-up, detailed treatment adherence monitoring, and external validation are warranted to reinforce our conclusions.

In conclusion, PCAD patients exhibit distinct coronary plaque vulnerabilities strongly linked to metabolic and lifestyle risk factors. OCT enables precise identification of high-risk features (e.g., thin caps, macrophage infiltration, microchannels), providing critical imaging evidence for early risk stratification and personalized intervention. Integrating artificial intelligence, multimodal imaging, and novel targeted therapies may advance comprehensive management from “plaque diagnosis” to “precision intervention”, ultimately reducing acute cardiovascular events.

## Data Availability

The original contributions presented in the study are included in the article/Supplementary Material, further inquiries can be directed to the corresponding authors.
